# Spatial-temporal targeting of lung-specific mesenchyme by a *Tbx4* enhancer

**DOI:** 10.1186/1741-7007-11-111

**Published:** 2013-11-13

**Authors:** Wenming Zhang, Douglas B Menke, Meisheng Jiang, Hui Chen, David Warburton, Gianluca Turcatel, Chi-Han Lu, Wei Xu, Yongfeng Luo, Wei Shi

**Affiliations:** 1 Developmental Biology and Regenerative Medicine Program, Department of Surgery, Children’s Hospital Los Angeles, Keck School of Medicine, University of Southern California, 4650 Sunset Blvd., MS 35, Los Angeles, CA 90027, USA; 2 Department of Genetics, University of Georgia, Athens, GA 30602, USA; 3 Department of Molecular and Medical Pharmacology, Geffen School of Medicine, University of California Los Angeles, Los Angeles, CA 90095, USA

**Keywords:** Lung mesenchyme, *Tbx4* lung enhancer, Tet-On system

## Abstract

**Background:**

Reciprocal interactions between lung mesenchymal and epithelial cells play essential roles in lung organogenesis and homeostasis. Although the molecular markers and related animal models that target lung epithelial cells are relatively well studied, molecular markers of lung mesenchymal cells and the genetic tools to target and/or manipulate gene expression in a lung mesenchyme-specific manner are not available, which becomes a critical barrier to the study of lung mesenchymal biology and the related pulmonary diseases.

**Results:**

We have identified a mouse *Tbx4* gene enhancer that contains conserved DNA sequences across many vertebrate species with lung or lung-like gas exchange organ. We then generate a mouse line to express rtTA/LacZ under the control of the *Tbx4* lung enhancer, and therefore a Tet-On inducible transgenic system to target lung mesenchymal cells at different developmental stages. By combining a Tbx4-rtTA driven Tet-On inducible Cre expression mouse line with a Cre reporter mouse line, the spatial-temporal patterns of *Tbx4* lung enhancer targeted lung mesenchymal cells were defined. Pulmonary endothelial cells and vascular smooth muscle cells were targeted by the Tbx4-rtTA driver line prior to E11.5 and E15.5, respectively, while other subtypes of lung mesenchymal cells including airway smooth muscle cells, fibroblasts, pericytes could be targeted during the entire developmental stage.

**Conclusions:**

Developmental lung mesenchymal cells can be specifically marked by *Tbx4* lung enhancer activity. With our newly created Tbx4 lung enhancer-driven Tet-On inducible system, lung mesenchymal cells can be specifically and differentially targeted *in vivo* for the first time by controlling the doxycycline induction time window. This novel system provides a unique tool to study lung mesenchymal cell lineages and gene functions in lung mesenchymal development, injury repair, and regeneration in mice.

## Background

The lung is originally developed from ventral foregut endoderm and surrounding splanchnic mesoderm [1,2]. Reciprocal interactions between lung mesenchymal and epithelial cells play essential roles in lung organogenesis and homeostasis. In fetal mice, lung epithelial cells are initially specified by Nkx2.1 expression around embryonic day (E) 9.5, followed by lung bud growth, airway branching morphogenesis, and terminal saccular formation [3]. During this developmental process, a wide variety of lung-specific epithelial cells are differentiated from their epithelial progenitor cells. The molecular markers and related animal models to target these epithelial cells are relatively well studied. However, developmental lung mesenchymal progenitor cells and their differentiation are poorly understood. Many unsolved issues of lung mesenchymal biology, such as whether mesenchymal cells in the developing lung are different from those in other organs and whether lung smooth muscle cells in airways and vasculature are derived from the same lung mesenchymal progenitors, remain critical questions in the field of lung research. Furthermore, no animal model is available to specifically target lung mesenchymal cells in order to manipulate gene expression in these cells [4]. Therefore, novel molecular approaches and genetic tools to specifically target lung mesenchyme from the beginning of lung formation are urgently needed.

Tbx4 is a member of the T-box transcription factor family, which play important roles during embryonic development through modulating gene expression [5]. Endogenous *Tbx4* gene expression is detected in many mesoderm-derived tissues including lung mesenchyme [6], but is not specific for lung [7]. However, Menke *et al*. recently reported that *Tbx4* expression in different tissues is controlled by a dispersed group of enhancers at different loci within the *Tbx4* genomic structure. One of these is located in the third intron and is conserved among several mammalian species [8]. A 5.5 kb DNA segment from this region is able to drive transgenic reporter expression in the developing lung and trachea at E12.5. However, detailed characteristics of this lung enhancer, including the spatial-temporal pattern of the enhancer activity at different developmental or post-developmental stages, are not known. By taking advantage of this potential lung-specific enhancer of the mouse *Tbx4* gene expression, we have generated a new *Tbx4* lung enhancer driven-reverse tetracycline transactivator (Tbx4-rtTA) transgenic mouse line. We then developed a lung-specific Tet-On inducible transgenic mouse model by crossing Tbx4-rtTA mice with TetO-Cre mice. Using *loxP*-mTomato-STOP-*loxP*-mGFP (mT-mG) reporter mice, we were thus able to identify and define the spatial-temporal pattern of Tbx4-rtTA-targeted lung mesenchymal progenitors and their derived cells during different stages of lung development and adulthood. Thus, our new lung mesenchymal-specific Tet-On inducible genetic system provides a valuable tool for the study of lung mesenchymal cells under both physiological and pathophysiological conditions.

## Results

### The lung enhancer of the mouse *Tbx4* gene contains genomic DNA sequence elements that are highly conserved across species that have lungs or lung-like gas exchange organs

**Figure 1:**
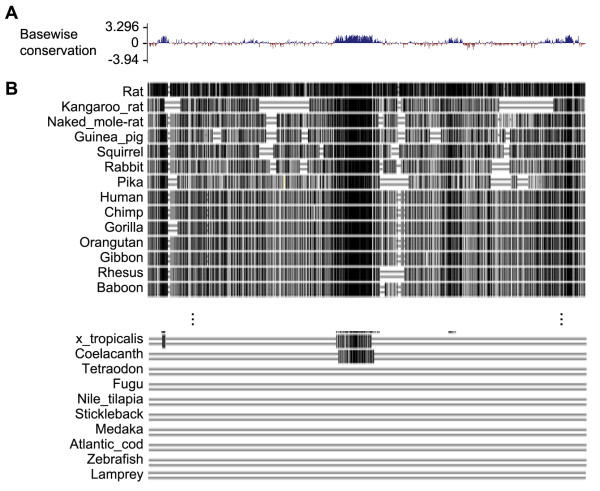
**Analysis of the 5.5 kb genomic DNA sequences of the potential mouse *Tbx4 *lung enhancer. (A)** Placental mammal basewise conservation scores determined by *phyloP* program [11]. Positive scores are assigned to the sites with conserved DNA sequences among the studied vertebrate species, which include mouse, rat, kangaroo rat, naked mole rat, guinea pig, squirrel, rabbit, pika, human, chimp, gorilla, orangutan, gibbon, rhesus, baboon, marmoset, squirrel monkey, tarsier, mouse lemur, bushbaby, treeshrew, pig, alpaca, dolphin, sheep, cow, cat, dog, panda, horse, microbat, megabat, hedgehog, shrew, elephant, rock hyrax, tenrecs, manatee, armadillo, sloth, opossum, Tasmanian devil, wallaby, platypus, turkey, chicken, zebra finch, budgerigar, lizard, painted turtle, xenopus tropicalis, coelacanth, tetraodon, fugu, nile tilapia, stickleback, medaka, Atlantic cod, zebrafish, lamprey. **(B)** The related sequence alignments of the above vertebrates to mouse Tbx4 genomic DNA sequence by Multiz program [12]. Most sequence alignments were omitted due to limited space. Identical nucleotide sequences are indicated by vertical black bar.

In order to understand the role of the 5.5 DNA fragment in mouse *Tbx4* intron 3 that has the potential to drive gene expression in mouse lung [8], the evolutionary conservation of this region was analyzed across 60 different vertebrate species using the BLAT program available through University of California Santa Cruz (UCSC) Genome Bioinformatics [9]. Interestingly, several fragments of the DNA sequences exhibited high similarity in 40 placental mammals (Figure 1A). In particular, a DNA fragment of about 500 bp in the middle of the region of interest appeared highly conserved in most vertebrate species that have a lung-like respiratory organ. For instance, coelacanth, an ancient kind of fish close to lungfishes and tetrapods, not only has a fat-filled single-lobed lung [10], but also a sequence homologous to this mouse *Tbx4* region. In contrast, no such DNA sequence similarity was found in other fishes that do not develop lung-like structures (Figure 1B). This suggests that this *Tbx4* DNA segment may be an early evolutionary adaptation that is important for successful morphogenesis of gas exchange organs for air breathing.

### Generation of a *Tbx4*-lung enhancer driven Tet-On system that specifically targets lung mesenchyme

**Figure 2:**
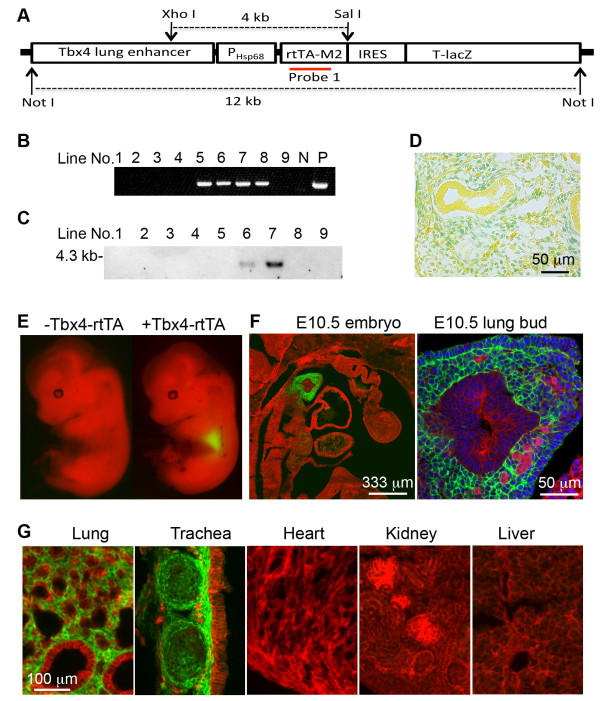
**Generation of an embryonic/fetal lung-specific Tbx4-rtTA transgenic mouse line. (A)** Schematic diagram of transgenic DNA construct. The position of the DNA probe for Southern blot is indicated. **(B)** Screen of transgenic founder lines by genomic DNA PCR. **(C)** Verification of transgenic lines by Southern blot. Genomic DNA was digested by XhoI and SalI prior to separation by gel electrophoresis. **(D)** Expression of rtTA-IRES-*LacZ* transgene in E13.5 lung was verified by X-gal staining (blue). **(E)** Cre-mediated mGFP expression (green) was detected in the lung of the triple transgenic mice (Tbx4-rtTA/TetO-Cre/mT-mG), but not in the double transgenic control mice (TetO-Cre/mT-mG), in which floxed-mTomato expression (red) was not affected. Dox induction was started from E6.5, and E13.5 mouse embryos were isolated and visualized under a fluorescence dissecting microscope. **(F)** Sagittal frozen section of E10.5 embryo of the triple transgenic mice with Dox induction from E6.5 to E10.5. Right panel shows the lung bud structure under high magnification. **(G)** Fluorescence microscopic examination of tissue frozen sections from E18.5 triple transgenic mice (Tbx4-rtTA/TetO-Cre/mT-mG), in which Dox induction was initiated from E6.5. mGFP was detected only in lung and tracheal mesenchyme, but not in other tissues. Dox, doxycycline; E, embryonic day.

Tbx4-rtTA transgenic mice were generated using a DNA vector as illustrated in Figure 2A, in which simultaneous expression of a reverse tetracycline transactivator (rtTA) and a *lacZ* (rtTA-IRES-*LacZ*) was controlled by the 5.5 kb mouse *Tbx4* lung enhancer. Two founder lines of mice were positive for Tbx4-rtTA transgene integration as detected by both genomic PCR and Southern blot (Figure 2B-C). Active expression of the transgene rtTA-IRES-*LacZ* driven by the *Tbx4* lung enhancer was easily verified by detecting nuclear LacZ activity with X-gal staining (Figure 2D). In order to further trace and characterize the cell types with *Tbx4*-driven rtTA expression, the mice were then crossed to TetO-Cre mice to make a Tet-On inducible Cre expression system [13], in which Cre expression was induced in rtTA expressing cells whenever doxycycline (Dox) is added. By further crossing to a mT-mG (*loxP*-mTomato-STOP-*loxP*-mGFP) fluorescent protein reporter mouse line [14], cells with Cre expression can be easily detected by mGFP expression as a result of Cre-mediated floxed-mTomato deletion, while Cre-negative cells still express mTomato. As shown in Figure 2E, Dox induction (E6.5 to E13.5) resulted in mGFP expression only in embryonic lung of the triple transgenic mice (Tbx4-rtTA/TetO-Cre/mT-mG) by gross view. In contrast, control mice without Tbx4-rtTA transgene (TetO-Cre/mT-mG) did not express mGFP, confirming the specificity of the Tbx4-rtTA transgene-mediated Cre reporter system. Moreover, sagittal sections of the E10.5 triple transgenic embryo showed that Cre-mediated mGFP expression was restricted to the lung buds and not detected in other organs. Within the lung bud, mGFP expression was restricted to mesenchymal cells surrounding the epithelial buds, including the outer layer of mesothelial progenitor cells of the visceral pleural membrane (Figure 2F). This lung/tracheal mesenchyme-specific pattern of Cre induction by the Tbx4-rtTA transgene persisted to the end of gestation, since no mGFP was detected in organs other than the lung and trachea of the triple transgenic mice with continuous Dox induction from E6.5 to E18.5 (Figure 2G).

### Dynamic pattern of *Tbx4* lung enhancer-driven lung mesenchymal cell targeting during and after development

**Figure 3:**
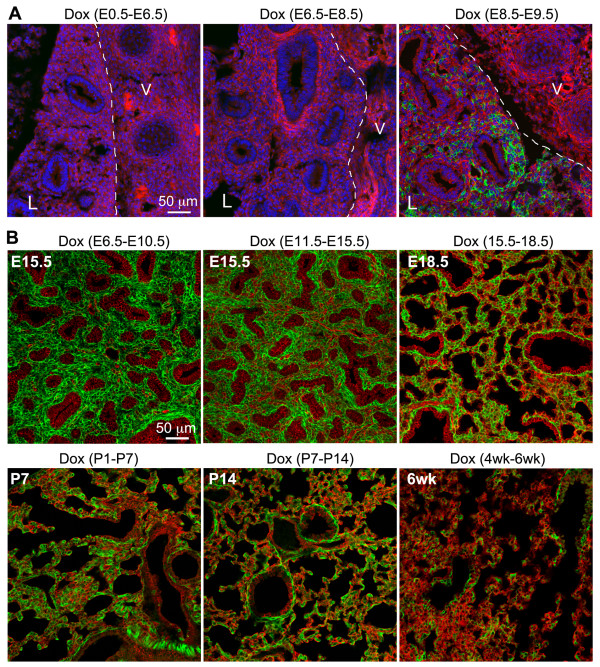
**Dynamic expression profile of Tbx4-rtTA-mediated Tet-On targeting system shown by the triple transgenic reporter (Tbx4-rtTA/TetO-Cre/mT-mG). (A)** Tbx4-rtTA-mediated Tet-On system targeted lung mesenchyme was initiated immediately after lung morphogenesis at E9.5. Dox induction was given at different time windows of early gestation, and the sagittal frozen sections of E13.5 embryos were used to detect mGFP (green) and mTomato (red) expression. Nuclei were counterstained with DAPI (blue). Lung tissue (L) was marked on the left side of the dotted line, and vertebrae (V) were located on the right side. **(B)** Efficiencies of cell targeting by the Tbx4-rtTA mediated Tet-On inducible system at different prenatal and postnatal stages. The time windows of Dox induction are indicated above each panel, and the ages of the examined lung specimens are specified inside each panel. DAPI, 4',6-diamidino-2-phenylindole; Dox, doxycycline; E, embryonic day.

As mentioned above, mouse lung development starts around E9.5. Based on the morphological features, lung development can be arbitrarily divided into pseudoglandular (E10.5-E16.5), canacular (E16.5-E17.5), saccular (E17.5-P5), and alveolar (P5-P30) stages. Lung mesenchymal progenitor cells proliferate and differentiate into different types of cells in concert with lung epithelial growth. Therefore, Tbx4-rtTA targeted lung mesenchymal cells may undergo developmental changes at different lung developmental stages as well as in post-development of the adult lung. Thus, the Tbx4-rtTA mediated Tet-On Cre inducible triple transgenic reporter mice were examined when Dox induction was initiated at different gestation time windows and postnatal ages. As shown in Figure 3A, Dox administration before lung formation (E8.5) was not able to induce Cre expression in any tissues including lung, as illustrated by negative mGFP expression in E13.5 embryos, while Dox administration from E8.5 to E9.5 started to induce Cre expression in some lung mesenchymal cells. During prenatal and postnatal lung development, the Tbx4-rtTA driven Tet-On system was very efficient in inducing Cre expression in lung mesenchyme (Figure 3B). For example, about 91% of cytokeratin-negative cells in peripheral lung saccular structure at E18.5 had inducible Cre expression when Dox was given to the triple transgenic mice (Tbx4-rtTA/TetO-Cre/mT-mG) from E6.5 to E18.5. Although Cre induction in adult lung was still detected in many lung mesenchymal cells including airway smooth muscle cells, the efficiency of Cre induction was reduced compared to that seen during lung development, as shown by reduction in the proportion of mGFP-positive cells (Figure 3).

### Differential targeting of smooth muscle cells by the *Tbx4* lung enhancer in developing lung

In the triple transgenic (Tbx4-rtTA/TetO-Cre/mT-mG) mouse system described above, mGFP expression persists within cells once Cre-mediated floxed-mTomato deletion has occurred. Thus, mGFP-positive cells do not necessarily represent an active status of the *Tbx4* lung enhancer-driven rtTA/LacZ expression. Since the TetO-Cre transgene is ubiquitously expressed, the Tet-On induction is dependent upon Tbx4-rtTA/LacZ transgenic expression. Therefore, by comparing the expression pattern of mGFP and LacZ in mouse lungs with different Dox induction time windows, cells that previously expressed Cre in early lung development could be distinguished from the cells that were still actively expressing Cre. Hence, the activity of *Tbx4* lung enhancer-mediated targeting to a variety of differentiated lung mesenchymal cells could be analyzed.

**Figure 4:**
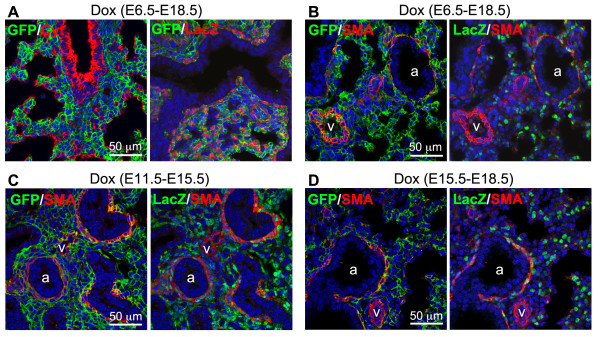
**Differential targeting of lung smooth muscle cells by the *Tbx4 *lung enhancer at different gestational ages. (A)***Tbx4* lung enhancer-mediated Tet-On induction activity was only detected in cytokeratin (Cyt)-negative cells, shown by GFP and Cyt co-immunostaining. In addition, not all GFP-positive cells were lacZ-positive, shown by GFP and LacZ co-immunostaining. The lung specimens were taken from the E18.5 triple transgenic fetuses with Dox induction time windows indicated above the panels. **(B-D)** Co-immunostaining of GFP, LacZ and SMA for the lungs of E18.5 **(B)**, E15.5 **(C)**, and E18.5 **(D)** triple transgenic fetuses with different Dox inductions indicated above the panels. a: airway; v: vasculature. Dox, doxycycline; SMA, α-smooth muscle actin.

With continuous induction of Dox during the entire period of fetal lung development (E6.5 to E18.5), no mGFP-positive epithelial cells were detected as demonstrated by GFP and cytokeratin co-immunostaining (Figure 4A). Furthermore, not all mGFP-positive cells were LacZ positive, while LacZ-positive cells were all mGFP-positive (Figure 4A), suggesting that some of the targeted lung mesenchymal cells lost the *Tbx4* lung enhancer activity during lung development. In order to understand whether loss of the *Tbx4* lung enhancer activity is related to changes in differentiation status of lung mesenchymal cells, we first looked at lung smooth muscle cells by examining co-expression of α-smooth muscle actin (SMA) and mGFP or SMA and LacZ. As shown in Figure 4B, Dox induction from E6.5 to E18.5 resulted in mGFP expression in both airway and vascular smooth muscle cells, suggesting that the smooth muscle cells of these structures were originally targeted by *Tbx4* lung enhancer during fetal lung development. However, LacZ expression was only detected in smooth muscle cells of the E18.5 airways, but not in the vascular smooth muscle cells, indicating that the *Tbx4* lung enhancer was not active in differentiated vascular smooth muscle cells at late gestation (E18.5). We then further determined the developmental time windows during which the *Tbx4-*lung enhancer was turned off in vascular smooth muscle cells. In E15.5 lung with Dox induction from E11.5, co-expression of mGFP and LacZ was seen in smooth muscle cells of both airway and vasculature; however, the intensity of lacZ staining in vascular smooth muscle cells was significantly reduced compared to that seen in airway smooth muscle cells. In contrast, in E18.5 lung with Dox induction from E15.5, co-expression of mGFP and LacZ was detected in airway smooth muscle cells only, with no expression of mGFP and LacZ in the vascular smooth muscle cells, suggesting that the *Tbx4*-lung enhancer was not active in differentiated vasculature smooth muscle cells after E15.5. Therefore, the *Tbx4* lung enhancer is able to target smooth muscle cells of both airway and vascular lineages prior to E15.5, but mainly airway smooth muscle cells at later times in gestation.

### Differential targeting of pulmonary endothelial cells by the *Tbx4* lung enhancer in developing lung

**Figure 5:**
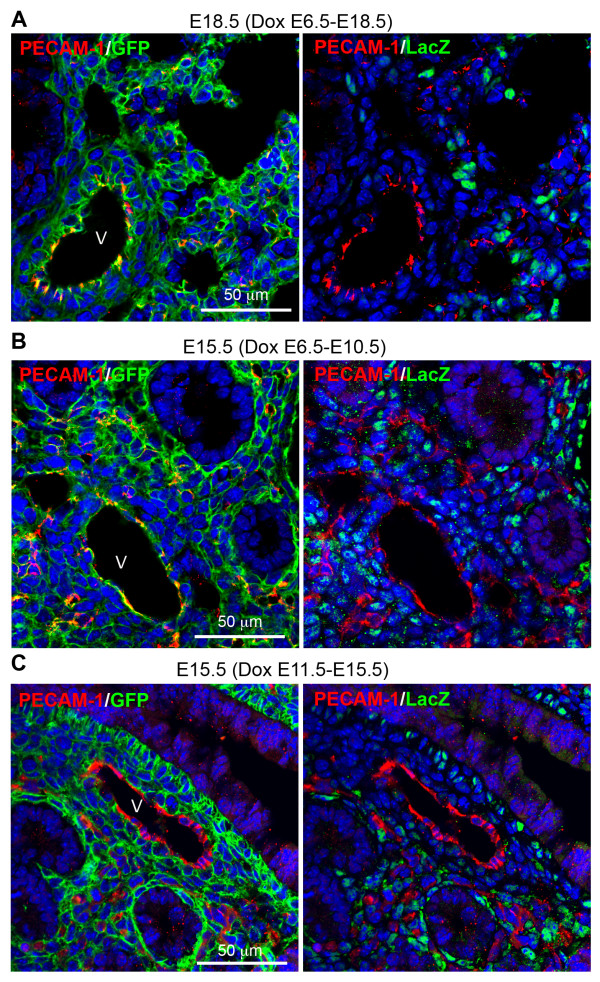
**Differential targeting of lung vascular endothelial cells by the *Tbx4 *lung enhancer at different gestational ages. (A)** In E18.5 lung of the triple transgenic mice with Dox induction from E6.5, PECAM-1-positive endothelial cells were GFP positive, but LacZ negative. **(B-C)** in the E15.5 lung of the triple transgenic mice with different Dox induction time windows as indicated above the panel, vascular endothelial cells were GFP-positive if Dox induction was given prior to E10.5 **(B)**, but LacZ expression was already negative. Consistently, Dox induction from E11.5 to E15.5 was not able to target vascular endothelial cells, shown by double negative staining for GFP and LacZ **(C)**. V: vasculature. Dox, doxycycline; E, embryonic day; PECAM-1, platelet endothelial cell adhesion molecule.

Endothelial cells in the pulmonary vasculature were also mGFP-positive in the triple transgenic lung specimens with Dox induction from E6.5 to E18.5 (Figure 5A). However, these mGFP-positive endothelial cells were LacZ-negative at E18.5, suggesting that the *Tbx4* lung enhancer might target endothelial progenitor cells during early lung morphogenesis. We then looked at lung specimens at different developmental stages in combination with different Dox induction time windows. In E15.5 triple transgenic lung with Dox induction from E6.5 to 10.5, endothelial cells, detected by platelet endothelial cell adhesion molecule (PECAM-1) immunostaining, were positive for mGFP, but not for LacZ (Figure 5B). Moreover, in E15.5 triple transgenic lung with Dox induction from E11.5 to 15.5, all PECAM-1-positive cells were negative for both mGFP and LacZ (Figure 5C). These data indicate that pulmonary endothelial cells may be derived from lung mesenchymal progenitors at an early gestation stage prior to E11.5 and that the *Tbx4* lung enhancer-mediated Tet-On system is able to target these progenitor cells if Dox induction is given before E11.5.

### Other lung cell lineages targeted by the *Tbx4* lung enhancer during lung development

**Figure 6:**
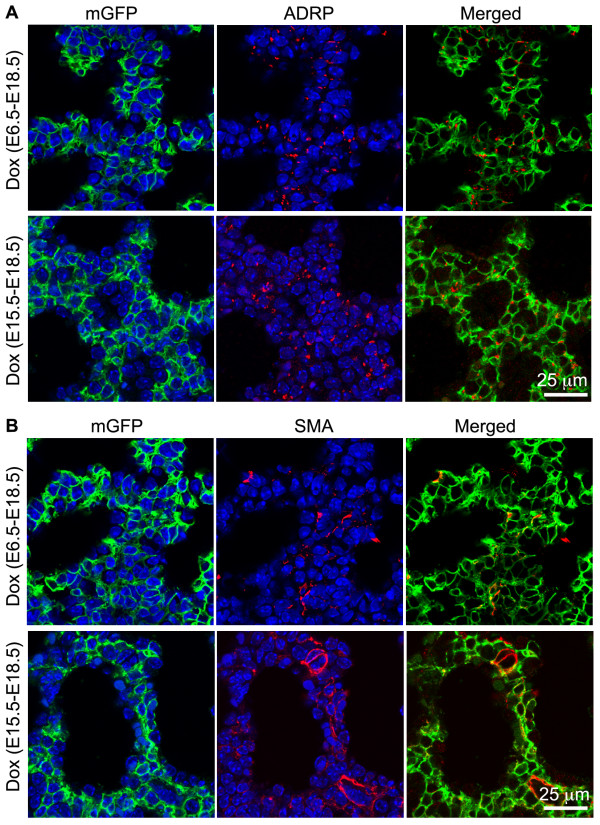
**Both myofibroblasts (SMA positive) and lipofibroblasts (ADRP positive) were marked by the *Tbx4 *lung enhancer driven Tet-On system.** E18.5 lung tissue sections of triple transgenic mice (Tbx4-rtTA/TetO-Cre/mT-mG) with different Dox induction during early or late gestation as indicated were co-stained with mGFP/ADRP **(A)** or mGFP/SMA **(B)**. Cell nuclei, counter-stained with DAPI (blue), were not included in the merged panels. ADRP, adipophilin; DAPI, 4',6-diamidino-2-phenylindole; Dox, doxycycline; E, embryonic day; SMA, α-smooth muscle actin.

**Figure 7:**
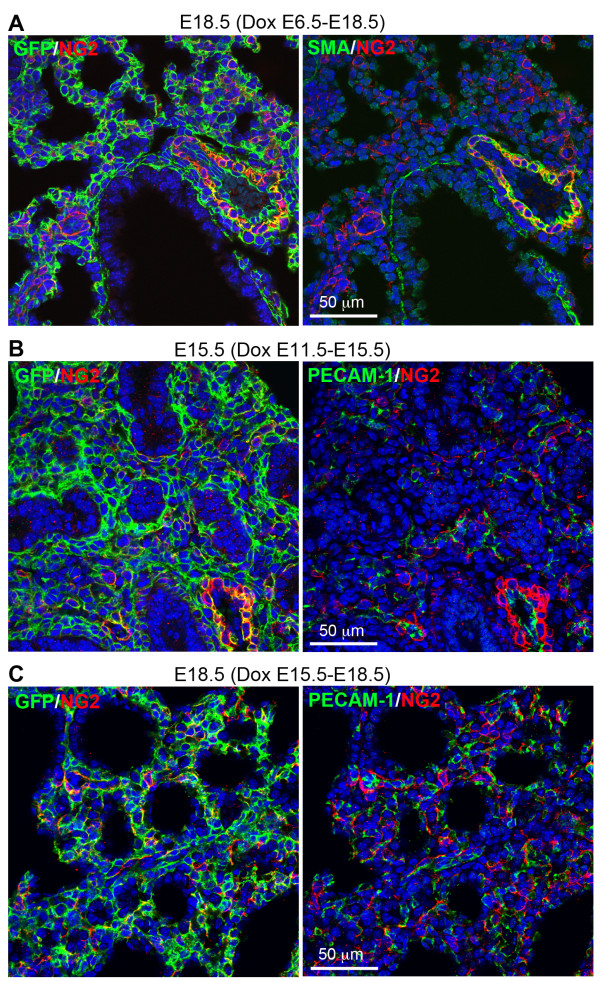
**NG2-positive cells were targeted by the *Tbx4*-lung enhancer driven Tet-On system.** Lung tissue sections of the triple transgenic mice (Tbx4-rtTA/TetO-Cre/mT-mG) with different Dox induction as indicated were co-immunostained by GFP, NG2, and SMA or PECAM-1. **(A)** In the E18.5 lung of the triple transgenic mice with Dox induction from E6.5 to E18.5, NG2-positive cells, including SMA-positive vasculature smooth muscle cells and SMA-negative pericytes, were all marked by GFP expression, suggesting that these cells were targeted by the Tet-On system in fetuses. **(B-C)** With Dox induction at either mid-gestation (E11.5 to E15.5) or late gestation (E15.5 to E18.5), most NG-2 positive cells of the triple transgenic mouse lungs at E15.5 **(B)** or E18.5 **(C)** were positive for GFP. These NG2-positive cells were adjacent to PECAM-1 positive endothelial cells. Dox, doxycycline; E, embryonic day; PECAM-1, platelet endothelial cell adhesion molecule; SMA, α-smooth muscle actin.

In addition to smooth muscle cells and endothelial cells, we also looked at other differentiated lung mesenchymal cells targeted by the *Tbx4* lung enhancer. Lung myofibroblasts and lipofibroblasts in the alveolar septal structures, which can be identified by SMA and adipophilin (ADRP) immunostaining, respectively [15,16], are important for lung alveolar formation and maintenance. In the primary alveolar sacs of E18.5 triple transgenic mice with Dox induction from either E6.5 to E18.5, or E15.5 to E18.5, both myofibroblasts and lipofibroblasts were all GFP-positive (Figure 6), suggesting that fetal Tbx4-rtTA expressing cells can be the progenitors for both of these specialized subpopulations of lung fibroblasts throughout the entire prenatal stage. Moreover, *Tbx4* lung enhancer-targeting was also detected in potential lung pericytes by co-immunostaining of GFP and NG2, one of the markers for pericytes (Figure 7A). In E15.5 lungs of the triple transgenic mice with Dox induction either from E6.5 to 10.5 or 11.5 to E15.5, NG2-positive staining was detected in both vascular smooth muscle cells and dispersed individual mesenchymal cells, which were all GFP-positive (Figure 7B-C). This suggests that the NG2-positive cells are derived from the *Tbx4* lung enhancer targeted cells. In contrast, pulmonary neuroendocrine cells, as identified by calcitonin gene-related peptide (CGRP) expression, were not targeted by the *Tbx4*-lung enhancer driver, as determined by negative GFP staining in the CGRP-positive cells of the triple transgenic mouse lung [see Additional file 1].

## Discussion

Mesenchymal cells in a variety of tissues are mainly derived from mesoderm during organogenesis. They play important roles in guiding organogenesis, generating the tissue-specific mesenchymal progenitor/stem cells needed for homeostasis in developed organs, and can differentiate into fibroblasts, smooth muscle cells, chondrocytes and other types of mesenchymal cells that support specific tissue functions. However, genetic markers specifically for mesenchymal cells in individual visceral tissue, including lung mesenchyme, have not been well defined. For example, in Dermo1-Cre knockin mice, *Dermo1* promoter drives Cre expression in many mesoderm-derived tissues including lung mesenchyme, diaphragm and ventral body walls [17-19]. Similarly, although endogenous *Tbx4* expression was detected in the mesenchyme of lung and trachea around E9.25, a few hours after the specification of the lung and trachea primordia by Nxk2.1 and Tbx5 [20], expression of both *Tbx4* and *Tbx5* genes is not restricted to lung tissue even though they are important in lung organogenesis [7]. Interestingly, a dispersed group of *Tbx4* gene enhancers are found to be responsible for the distinct tissue locations of this gene [8]. Among these, a 5.5 kb fragment of genomic sequences in *Tbx4* intron 3 is related to its expression in early embryonic lung. Our DNA sequence analysis suggests that several DNA fragments in this region, particularly an approximately 500 bp fragment in the middle region, are highly conserved in vertebrate species that develop lungs or lung-like gas exchange structures. This suggests that this DNA regulatory element may be important for lung structure formation. However, further experiments to examine individual fragments of the mouse *Tbx4* genomic region and compare their gene regulatory activities among different specimens will be needed to understand fully the role of mouse *Tbx4* lung enhancer in lung morphogenesis. Furthermore, whether these conserved fragments of DNA sequences in each species are involved in regulating *Tbx4* gene expression also needs to be experimentally analyzed.

We have used this lung-specific DNA enhancer of *Tbx4* gene to generate a Tet-On inducible transgenic system in mice. In combination with Cre-mediated reporter mice, we have clearly demonstrated that developing lung mesenchymal cells can be specifically marked from the beginning of lung formation, which makes these cells distinguishable from the mesenchymal cells arising from other organs. We have shown for the first time that lung mesenchymal cells can be targeted in an organ-specific manner by using the *Tbx4* lung enhancer, rather than the entire endogenous Tbx4 promoter as in the Tbx4-Cre knockin model [7]. This provides a powerful genetic tool with which to study the functions of genes in lung mesenchymal development, and to isolate and trace lung mesenchymal cell lineages during prenatal development through postnatal development to adulthood. More importantly, it provides the potential to generate unique mouse models mimicking lung interstitial diseases without adversely affecting other organs and systems. The molecular mechanisms by which the *Tbx4* lung enhancer is activated specifically in lung mesenchymal cells are not yet completely clear, and also will need further investigation. It is possible that specific lung mesenchymal cells may express a unique array of transcription factors that interact and activate the *Tbx4* lung enhancer. In contrast, mesenchymal cells in other tissues may lack some of these specific transcriptional activators, resulting in suppression of this particular *Tbx4* gene regulatory element, but not endogenous *Tbx4* gene expression that can be activated by multiple regulatory elements. Furthermore, our experiments also show that the *Tbx4* lung enhancer is not persistently active in all lung mesenchymal cells. Its activity is turned off in some committed and/or differentiated mesenchymal cell lineages at various developmental stages, particularly in pulmonary endothelial cells and vascular smooth muscle cells. Whether active *Tbx4* lung enhancer activity is related to lung mesenchymal progenitor cells or is associated with cell differentiation status remains to be determined.

Another feature of our Tbx4-rtTA-mediated Tet-On Cre inducible system is the combination of the Cre-mediated reporter and LacZ reporter systems. Cre-mediated mGFP expression is the marker for cells that had and/or have induced Cre expression, while LacZ expression indicates active *Tbx4* lung enhancer activity at the time of examination. Therefore, using this system, we have been able to determine the dynamic changes and fates of lung mesenchymal cells with *Tbx4* lung enhancer activity. In early lung buds, the majority of these mesenchymal progenitors can be marked using this *Tbx4* lung enhancer, and these cells are able to differentiate to endothelial cells and smooth muscle cells of both airway and vasculature. However, the multi-potent differentiation potential of these mesenchymal progenitor cells is reduced during the course of lung development, as the majority of endothelial cells and vascular smooth muscle cells are neither labeled by Cre-mediated mGFP expression if Dox induction is given after E11.5 and E15.5, respectively, nor detected for LacZ expression. However, lung airway smooth muscle cells and subtypes of fibroblasts (lipofibroblast and myofibroblast) are positive for induced mGFP expression even if Dox is given at late gestation. Therefore, differential targeting of mouse lung mesenchymal progenitors and related cell lineages can be achieved by controlling the time windows of Dox induction.

Interestingly, a proportion of mesenchymal cells in postnatal developing and even adult lung are still active for the *Tbx4*-lung enhancer. These cells are located adjacent to alveolar epithelial cells and alveolar endothelial cells of adult lung alveolar structure, and are positive for NG2 or SMA staining [see Additional file 2], suggesting that these may be pericytes and myofibroblasts. Therefore, our *Tbx4*-lung enhancer driven targeting driver line has the potential to be used in the study of adult lung diseases, such as asthma, emphysema and interstitial pulmonary fibrosis. It could be used to create new disease models, to determine the response of targeted cell lineages to specific injuries and/or to test intervention approaches to prevent or attenuate pathological processes. Moreover, understanding developmental lung mesenchymal specificity is also important when considering the future design of cell-based therapies. For example, the role of bone marrow-derived mesenchymal stem cells in lung injury repair and regeneration is controversial, although their effects on modulating immune and inflammatory responses are well recognized [21]. This raises the question whether mesenchymal stem cells derived from lung may function better in repairing lung structures than those originating from other organs such as bone marrow [22]. Our newly generated Tet-On system will make it possible to compare mesenchymal stem cells of lung with those of non-lung origins in mice by marking developing lung mesenchymal cells with prenatal Dox induction. Comparison of these two groups of cells and characterization of their molecular signatures under both physiological and pathological conditions will greatly advance our knowledge about lung biology and pulmonary diseases.

## Conclusions

Although mesenchymal cells in developing lung display complicated heterogeneous phenotypes, they are probably derived from splanchnic mesoderm surrounding lung progenitors in foregut endoderm. We have found an evolutionally conserved *Tbx4* lung enhancer that is active specifically in lung mesenchymal progenitors and subsets of their derived cells. Therefore, with our newly created *Tbx4* lung enhancer-driven Tet-On inducible system, lung mesenchymal cells can be specifically and differentially targeted for the first time by controlling the doxycycline induction time window. This novel system provides a unique tool to study lung mesenchymal cell lineages and gene functions in lung mesenchymal development, injury repair, and regeneration in mice.

## Methods

### DNA vector construction

rtTA2^s^-M2 DNA fragment was amplified from pTet-On Advanced vector (Clontech, Mountain View, CA, USA) using primers 5′-CGGCCCCGAATTCACCATGTCTAGA-3′ and 5′-ACGCGTCGACACTTAGTTACCCGGGGAGCATG-3′, and subcloned to EcoRI/SalI digested pBluescript KS II to generate pBS-rtTA. A 5.5 kb DNA fragment of mouse *Tbx4* lung enhancer and a 0.9 kb DNA fragment of HSP68 minimal promoter were obtained by NotI and SmaI/SfoI digestion of pDBM3 [8], respectively, and subcloned into pBS-rtTA to generate pBS-Tbx4-rtTA. Finally, IRES-T-LacZ DNA fragment was obtained by digesting pNTR-lacZ-PGKNeolox plasmid provided by Dr. Vesa Kaartinen at University of Michigan, and inserted to pBS-Tbx4-rtTA to produce an intact transgenic vector pBS-Tbx4-rtTA-LacZ. The prokaryotic part of the vector was then removed by NotI digestion and the 12 kb transgenic DNA fragment was isolated for C57BL/6 pronuclear injection.

### Mouse strains, breeding, and genotyping

Tbx4-rtTA mouse line was generated at the University of California at Los Angeles (UCLA) Transgenic Core facility. The founders were identified by genomic DNA PCR using primers 5′-GGA AGG CGA GTC ATG GCA AGA-3′ and 5′-AGG TCA AAG TCG TCA AGG GCA T-3′. The transgenic mice were further verified by DNA Southern blot for genomic DNA digested with XhoI and SalI. DIG-labeled nonradioactive detecting system (Roche, Indianapolis, IN, USA) was used.

The TetO-Cre mouse line was originally provided by Dr. Jeffrey Whitsett at Cincinnati Children’s Hospital [23,24]. mT-mG double fluorescent Cre reporter mice were obtained from Jackson Laboratory (Bar Harbor, ME, USA) [14]. All mice were bred in C57BL/6 background. Fetal lung mesenchymal-specific Cre expression was induced by Dox administration from different gestational stages by feeding the pregnant mice with both Dox food (625 mg/kg; TestDiet, Richmond, IN, USA) and Dox water (0.5 mg/ml; Sigma, St. Louis, MO, USA). In postnatal pups, Cre induction was initiated by a single intraperitoneal injection of Dox (100 mg/kg body weight) followed by Dox oral administration, the same as described above for the entire induction period. All reported mouse studies were approved by the Institutional Animal Care and Use Committee at the Saban Research Institute of Children’s Hospital Los Angeles.

### Fluorescence protein and immunofluorescence detection

Mouse embryos or lung tissues were dissected. For mT and mG detection, mouse embryos or tissues were directly imaged under a Leica MZFLIII fluorescence dissecting microscope. The embryos or tissues were then fixed with 4% buffered paraformaldehyde and embedded either in optimal cutting temperature (OCT) compound for frozen section or in paraffin for regular histological section. Tissue frozen sections were washed in PBS, and mounted with VECTASHIELD medium with DAPI (Vector Laboratories, Burlingame, CA, USA). Immunofluorescence staining was performed following methods published previously [25]. The related antibodies were: rabbit anti-GFP (Santa Cruz Biotechnology, Santa Cruz, CA, USA), goat anti-GFP (Abcam, Cambridge, MA, USA), mouse anti-cytokeratin and mouse anti-SMA (Sigma), rabbit anti-PECAM-1 (LSBio, Seattle, WA, USA), rabbit anti-lacZ (MP Biomedicals, Solon, OH, USA), mouse anti-ADRP (BioGenex, Fremont, CA, USA), rabbit anti-NG2 chondroitin sulfate proteoglycan (Millipore, Billerica, MA, USA), hamster anti-T1α (DSHB at the University of Iowa) and mouse anti-CGRP (Sigma). Fluorescent signals were detected using a Zeiss LSM710 confocal microscope at the Imaging Core Facility of the Saban Research Institute of Children’s Hospital Los Angeles. All experiments were repeated at least five times and data represent consistent results.

### X-gal staining

Lung specimens were briefly fixed with 4% paraformaldehyde in PBS containing 2 mM MgCl2 and embedded in OCT. Lung frozen sections were incubated with X-gal reaction buffer following the procedures described in a previous publication [17].

## Abbreviations

ADRP: Adipophilin; bp: Base pair; CGRP, Calcitonin gene-related peptide; Cyt: Cytokeratin; Dox: Doxycycline; E: Embryonic day; GFP: Green fluorescent protein; mT-mG: *loxP*-mTomato-STOP-*loxP*-mGFP; OCT: Optimal cutting temperature; P: Postnatal day; PBS: Phosphate-buffered saline; PCR: Polymerase chain reaction; PECAM-1: Platelet endothelial cell adhesion molecule; rtTA: Reverse tetracycline transactivator; SMA: α-smooth muscle actin.

## Competing interests

The authors declare that they have no competing interests.

## Authors’ contributions

Concept and design, WS; acquisition of data, WZ, DBM, HC, MJ, GT, CHL, WX, YL, WS; analysis and interpretation, WZ, DBM, MJ, DW, WS; drafting and editing of the manuscript, WZ, DW, WS. All authors read and approved the final manuscript.

## Supplementary Material

Additional file 1 Lung neuroendocrine cells were not targeted by the Tbx4 lung enhancer, shown by co-immunostaining of GFP (green) and CGRP (red) for E18.5 lung tissue section of the triple transgenic mouse (Tbx4-rtTA/TetO-Cre/mT-mG) with Dox induction from E6.5 to E18.5. Blue: DAPI nuclear counterstaining. Click here for file

Additional file 2 **Cells with Tbx4 lung enhancer activity in adult lungs. (A)** Adult mice with triple transgenic genotypes (Tbx4-rtTA/TetO-Cre/mT-mG) were induced by Dox administration for two weeks. The lung tissue sections were co-immunostained with GFP and one of the cell markers as indicated. Cells with GFP expression (green) were not positive for T1α and PECAM-1 (red). However, most GFP-positive cells were positive for NG2, and a few GFP-positive cells were positive for SMA, shown by overlapped co-staining (yellow color). DAPI was used for nuclear counterstaining (blue). **(B)** Evaluation of adult transgenic reporter mouse lungs in the absence of Dox induction. Mouse genotypes are indicated above the panel. Expression of endogenous mTomato (red) and mGFP (green) was examined for the lung frozen sections under fluorescence microscope. DAPI was used for nuclear counterstaining (blue). Click here for file
