# Supplementary material for: Spatial-temporal targeting of lung-specific mesenchyme by a Tbx4 enhancer
Source: BMC Biol. 2013 Nov 13;11:111. doi: 10.1186/1741-7007-11-111 (PMC3907025; doi:10.1186/1741-7007-11-111)
Supplement: Additional file 1 — Lung neuroendocrine cells were not targeted by the Tbx4 lung enhancer, shown by co-immunostaining of GFP (green) and CGRP (red) for E18.5 lung tissue section of the triple transgenic mouse (Tbx4-rtTA/TetO-Cre/mT-mG) with Dox induction from E6.5 to E18.5. Blue: DAPI nuclear counterstaining. [file 1741-7007-11-111-S1.pdf]

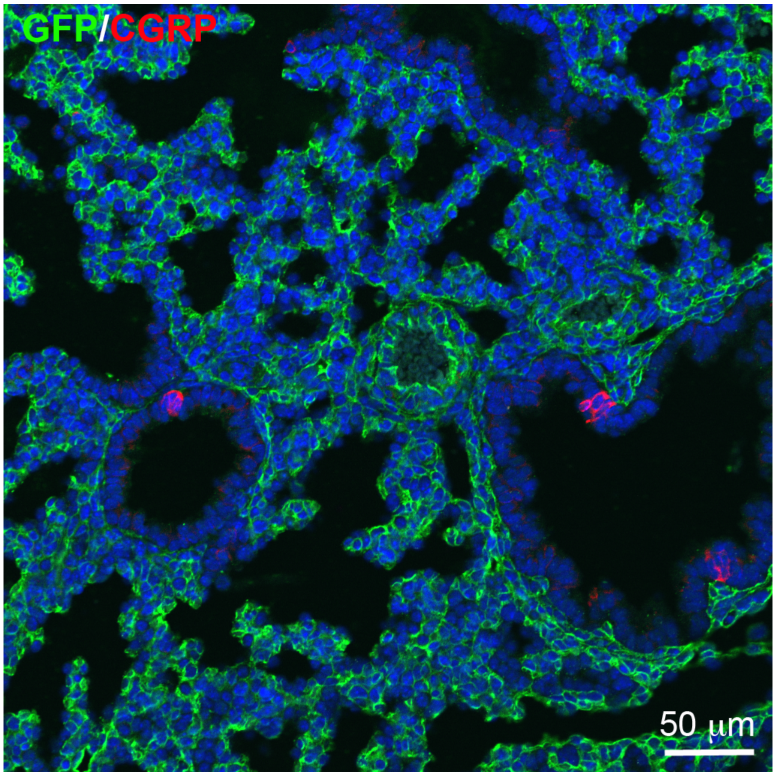

Additional file 1: Lung neuroendocrine cells were not targeted by the Tbx4 lung enhancer, shown by co-immunostaining of GFP (green) and CGRP (red) for E18.5 lung tissue section of the triple transgenic mouse (Tbx4-rtTA/TetO-Cre/mT-mG) with Dox induction from E6.5 to E18.5. Blue: DAPI nuclear counterstaining.
