# Supplementary material for: Spatial-temporal targeting of lung-specific mesenchyme by a Tbx4 enhancer
Source: BMC Biol. 2013 Nov 13;11:111. doi: 10.1186/1741-7007-11-111 (PMC3907025; doi:10.1186/1741-7007-11-111)
Supplement: Additional file 2 — Cells with Tbx4 lung enhancer activity in adult lungs. (A) Adult mice with triple transgenic genotypes (Tbx4-rtTA/TetO-Cre/mT-mG) were induced by Dox administration for two weeks. The lung tissue sections were co-immunostained with GFP and one of the cell markers as indicated. Cells with GFP expression (green) were not positive for T1α and PECAM-1 (red). However, most GFP-positive cells were positive for NG2, and a few GFP-positive cells were positive for SMA, shown by overlapped co-staining (yellow color). DAPI was used for nuclear counterstaining (blue). (B) Evaluation of adult transgenic reporter mouse lungs in the absence of Dox induction. Mouse genotypes are indicated above the panel. Expression of endogenous mTomato (red) and mGFP (green) was examined for the lung frozen sections under fluorescence microscope. DAPI was used for nuclear counterstaining (blue). [file 1741-7007-11-111-S2.pdf]

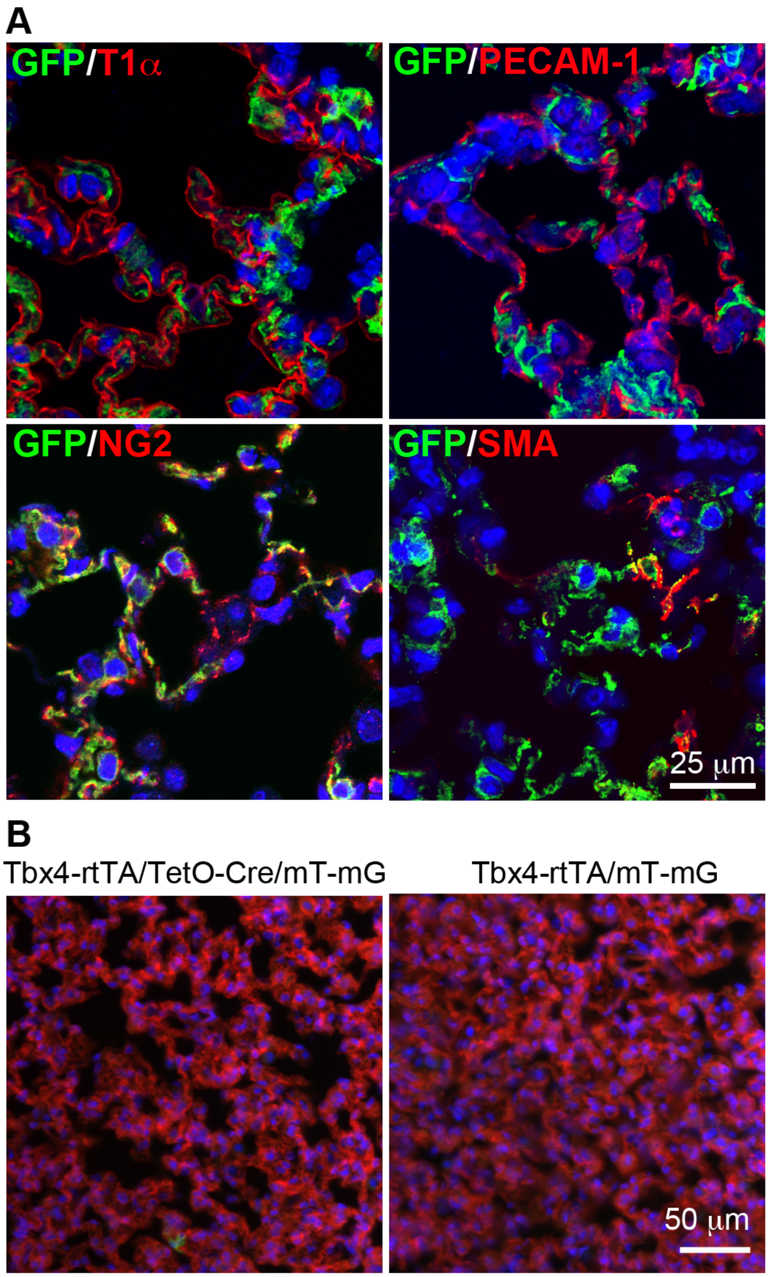

Additional file 2: Cells with Tbx4 lung enhancer activity in adult lungs. (A) Adult mice with triple transgenic genotypes (Tbx4-rtTA/TetO-Cre/mT-mG) were induced by Dox administration for 2 weeks. The lung tissue sections were co-immunostained with GFP and one of the cell markers as indicated. Cells with GFP expression (green) were not positive for T1α and PECAM-1 (red). However, most GFP-positive cells were positive for NG2, and a few GFP-positive cells were positive for SMA, shown by overlapped co-staining (yellow color). DAPI was used for nuclear counterstaining (blue). **(B)** Evaluation of adult transgenic reporter mouse lungs in the absence of Dox induction. Mouse genotypes are indicated above the panel. Expression of endogenous mTomato (red) and mGFP (green) was examined for the lung frozen sections under fluorescence microscope. DAPI was used for nuclear counterstaining (blue).
